# Effects of Er, Cr: YSGG Laser on Shear Bond Strength of the Orthodontic Brackets for 5 and 10 Seconds: An In Vitro Study

**DOI:** 10.1155/2022/9126699

**Published:** 2022-03-27

**Authors:** Ahmed A. Bagwan, Amal H. Abuaffan, Ali Alrahlah, Talat Hassan AL-Gunaid

**Affiliations:** ^1^Department of Orthodontics, School of Dentistry, University of Khartoum, Khartoum, Sudan; ^2^Department of Orthodontics, Pedodontics and Preventive Dentistry, University of Khartoum, Khartoum, Sudan; ^3^Department of Restorative Dental Sciences, College of Dentistry, King Saud University, Engr. Abdullah Bugshan Research Chair for Dental and Oral Rehabilitation, Riyadh 11545, Saudi Arabia; ^4^Department of Pediatric Dentistry and Orthodontics, College of Dentistry, Taibah University, Madinah, Saudi Arabia

## Abstract

**Objectives:**

The study designed to compare the effect of erbium, chromium: yttrium-scandium-gallium-garnet (Er, Cr: YSGG) laser at different power outputs and short periods of time (5 and 10 s) and acid etching on the shear bond strength (SBS) and failure mode of metallic orthodontic brackets. *Material and methods*. A total of sixty-nine human premolars extracted for orthodontic purposes were used. 60 teeth were randomly assigned to one of the five treatment groups. (*n* = 12): group 1: 37% phosphoric acid etching and groups 2–5: the enamel surface irradiated by the Er, Cr: YSGG laser operated at different power outputs (1 W, 1.5 W, 2 W, and 2.5 W), each laser group was divided into two subgroups (*n* = 6) according to exposure time (5 s and 10 s). Nine teeth were not subjected to SBS testing but were prepared for scanning electron microscopy (SEM). The nonparametric Kruskal–Wallis test was used to evaluate the data; the SBS and adhesive remanent index (ADI) were evaluated.

**Results:**

The mean SBS for all laser groups and the acid-etched group were comparable, with no significant differences except for the 1 W group for 5 and 10 s and the 1.5 W group for 5 s. For the ARI scores, no statistically significant difference was found among the groups (*P*=0.059), and the majority of the samples had ARI type 2 or 3.

**Conclusion:**

The laser irradiation at 2 and 2.5 W for 5 s was similar to that produced by acid etching, whereas the laser irradiation at 2 and 2.5 W at 10 s was higher compared with that obtained with acid etching and adequate to etch the enamel.

## 1. Introduction

Bond strength of orthodontic brackets has been studied extensively, with a wide range of data and publications available. The ideal orthodontic bond should ensure that the bracket remains attached to the tooth surface during the treatment, withstanding force application to achieve tooth movement and functional forces, and at the end of treatment, the attachment should be easily removed without damage to the tooth surface [1]. The bonding of brackets is formed by the adhesive mechanically fitting into the microporosities of the enamel surface. As a result, an effective bond necessitates precise enamel surface preparation [2].

The use of varying concentrations of phosphoric acid to induce microporosity on the enamel surface is a common pretreatment method. Microporosity aids in the creation of the micromechanical bond at the enamel-sealant interface [3]. However, phosphoric acid dissolves the mineral ions in the enamel layer, leaving it vulnerable to caries attack, especially in the presence of orthodontic attachments [4].

Hence, the researchers have been looking for alternative conditioning methods to overcome the shortcomings of acid etching for a long time. Lasers such as diode, CO_2_, Nd: YAG laser, and recently Er: YAG and Er, Cr: YSGG lasers are one of the most important technologies that may be used in a variety of fields, including medicine. Lasers have been employed in dentistry, including conservative dentistry [5], endodontics [6], periodontology [7], implantology [8], oral surgery [9], etc. It can be used in conditioning the teeth without forming a smear layer, remove the carious process with minimal invasiveness, create a microretentive surface for adhesive restorations, and influence the total microbial load reduction [10].

The Er, Cr: YSGG laser has 2780 nm wavelength and is absorbed strongly by both water and hydroxyapatite. The sudden evaporation of bound water causes microexplosions that blast away tiny particles of the tooth [11]. Furthermore, laser etching of enamel or dentin reportedly yields an anfractuous surface and open dentin tubules, which are ideal for adhesion [12]; thus, this method can be used as an alternative to acid etching of enamel and dentin [13–16].

The previous studies [13, 14, 17, 18] found that the optimal time for etching enamel with the Er, Cr: YSGG laser is 15 s, which is similar to phosphoric acid etching time. The purpose of our study was to investigate the shear bond strength, facial surface irregularities, and adhesive remnant scores over short time periods (5 s and 10 s).

The null hypotheses that there is no difference in the shear bond strength obtained by ER: CR, YSGG laser irradiation at 5 and 10 s and phosphoric acid 37% etching at 15 s.

## 2. Material and Methods

The ethical approval for this study was obtained from the “Institutional Ethical Committee” Faculty of Dentistry, University of Khartoum (Ref. No. 20191101).

### 2.1. Sample Grouping and Preparation

Sample size estimation was calculated using the *G*^*∗*^ Power software program, version 3.1.9.2, with error (*P* value) = 0.05 (95% confidence interval) and *β*-error (power of the study) = 95%, a total of 60 sound human premolars were required [19].

Sixty-nine intact human premolar teeth extracted for orthodontic requirement were used in the current study. The teeth were stored in 0.1% thymol solution at room temperature until use to prevent bacterial growth [20]. The teeth were visually examined and found to be free of caries, macroscopic fractures, and wear.

**Figure 1:**
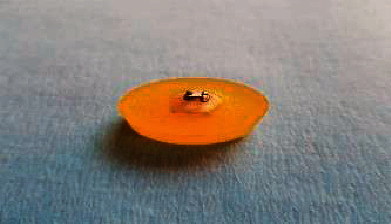
Tooth embedded in a holder ring.

All the teeth's crowns were sectioned from their roots. A chemically activated acrylic resin was used to implant the tooth crown horizontally in the specimen holder ring, leaving 2 mm of buccal enamel exposed (Figure 1). The enamel surfaces were pumiced, rinsed for 30 s, and dried for 10 s.

Afterward, 60 teeth were divided into five groups (*n* = 12) and were used in SBS testing as follows:

  Group 1: control group etched with 37% phosphoric acid
  Group 2: enamel conditioned with the Er, Cr: YSGG laser at 1 W power
  Group 3: enamel conditioned with the Er, Cr: YSGG laser at 1.5 W power
  Group 4: enamel conditioned with the Er, Cr: YSGG laser at 2 W power
  Group 5: enamel conditioned with the Er, Cr: YSGG laser at 2.5 W power Table 1

Each laser group was divided into two subgroups (*n* = 6) according to exposure time (5 s and 10 s) (Table 2).

The remaining nine teeth (one specimen for each subgroup) were utilized for scanning electron microscopy (SEM) inspection to assess the topography and morphology of the treated enamel.

### 2.2. Etching by Phosphoric Acid

The control group consisted of 12 premolars etched for 15 s using an orthophosphoric acid gel (META Etchant, Meta Biomed, Korea). The teeth were cleaned and dried. The enamel in all etched teeth appeared chalky white.

### 2.3. Laser Treatment

**Figure 2:**
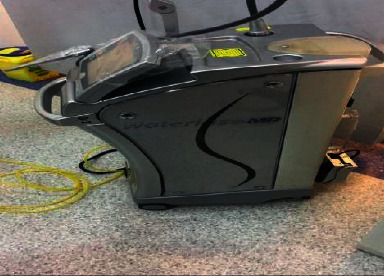
Er, Cr: YSGG laser device.

**Figure 3:**
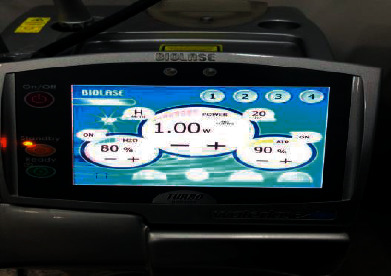
At 80% water and 90% air.

The Er, Cr: YSGG laser system (WaterLase iPlusTM, Biolase Inc., USA) was used to irradiate the laser groups and adjusted to 80% water and 90% air, 2.78 *μ*m wavelength, 20 Hz frequency, and 140 *μ*s pulse duration [21] (Figure 2). A fiberoptic system sent laser energy to a sapphire tip terminal with a diameter of 600 *µ*m and a length of 6 mm. The beam was swept across the enamel perpendicularly at a distance of 1–1.5 mm. Only the power output of the laser system was changed, but the wavelength remained constant. Despite that the output power can be varied from 0 W to 6 W, the four power values used in this study were 1, 1.5, 2, and 2.5 W (Figure 3).

### 2.4. Bonding Procedure

The bonding procedure in the laser-irradiated and acid-etched groups followed the manufacturer's instructions. Stainless steel premolar brackets with a surface area of 10 mm^2^ (American Orthodontic Co., Washington Avenue, Sheboygan, Wisconsin, USA) were used in this study and bonded by an adhesive paste (Transbond XT Light Cure adhesive primer and Transbond XT adhesive resin, 3M Unitek, Monrovia, California, USA). The braces were placed firmly on the tooth surface and cured with mini-LED (Acteon, France) for 40 s in the mesial, distal, occlusal, and gingival aspects (10 s each); then, they were placed in distilled water at 37°C for 24 hours.

To imitate the heat and humidity conditions of the oral cavity, all specimens were placed in an automatic thermocycling apparatus (SD Mechatronik Thermocycler, SD Mechatronik GmbH, Feldkirchen-Westerham, Germany) with water baths maintained at 5°C and 55°C for 30 s cycles, for a total of 500 cycles [22]. After 2 days of storage at room temperature in distilled water, a shear bonding test was performed.

### 2.5. Shear Bond Strength (SBS) Testing

**Figure 4:**
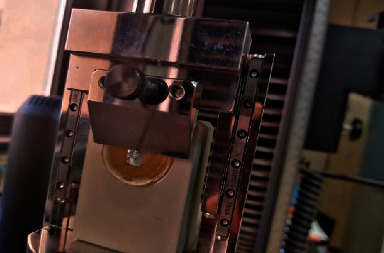
Force applied by the universal testing machine tapered blade between the bracket base and the tooth.

The 60 bonded brackets underwent SBS testing by using a chisel edge mounted on the crosshead of a testing machine (Model 5565, MA, USA) and loaded to failure under compression using a knife-edge loading head at a crosshead speed of 0.5 mm/min; the SBS was expressed in megapascals (MPa), which was derived by dividing the imposed force (Newtons) by the bracket base area (mm^2^) (Figure 4).

### 2.6. Scanning Electron Microscopy (SEM)

The remaining nine unbonded teeth (treated by acid etch and laser) underwent SEM evaluation (JEOL JSM 6360, Tokyo, Japan) at an accelerating voltage 20 kV to determine whether the phosphoric acid-etched and laser-irradiated teeth had differences in surface quality. The SEM teeth were inspected according to the criteria of Silverstone et al. [23] and Galil and Wright [24].

  Type 1: honeycomb image because of dissolved central part of enamel prism
  Type 2: pebble image because of dissolved periphery of enamel prism
  Type 3: type 1 and 2 images together
  Type 4: reticular and uncomplicated pattern and similar map image of the enamel surface
  Type 5: flat and smooth image of the enamel surface

### 2.7. Residual Adhesive

The teeth after debonding were examined with 10× magnification using a stereomicroscope (model SMZ 1000, Nikon, Tokyo, Japan) equipped with a digital camera (model DXM1200, Nikon) and image software to determine the quantity of residual adhesives remaining on each tooth (Nikon ACT-1, version 2.62, Nikon). The adhesive remnant index ARI [25] was created to quantify the amount of adhesive using a scale ranging from 0 to 3, as follows:

(0) No composite is left on the enamel
(1) Adhesive covers less than half of the enamel bonding site
(2) Adhesive covers more than half of the enamel bonding site
(3) Adhesive totally covers the enamel bonding site

### 2.8. Statistical Analysis

The collected data were entered in a spread sheet (Microsoft excel), double checked, and transferred to the statistical package for the social sciences software program for Windows (SPSS v25, IBM Corp) for further analysis. The results were presented as means and standard deviations for SBS and frequencies and percentages for ARI. The differences in SBS between the different powers (*W*) and times (5 and 10 s) were utilized using the nonparametric Kruskal–Wallis test. If significant, a further multiple comparison test was performed. The associations between ARI, acid etching, and laser etching groups were determined using the chi-squared test. A *P* value of less than 0.05 was considered significant for all tests.

## 3. Results

### 3.1. Shear Bond Strengths

Table 3 summarizes the descriptive statistics for the SBS of all groups, regardless of time. The lowest SBS mean value of the laser etching groups was found in the 1 W group (3.30 ± 1.69 MPa); the highest SBS mean value of 11.39 ± 6.32 MPa was found in the 2 W group. The SBS mean value of the acid etching group was 10.28 ± 5.12 MPa.

Table 4 summarizes the descriptive statistics for the SBS of the laser groups based on exposure times. The lowest SBS mean value of the laser etching group (5 s) was found in the 1 W group (2.31 ± 0.85 MPa), and the highest SBS mean value of 10.64 ± 8.70 MPa was found in the 2 W group. Similarly, the lowest SBS mean value of the laser etching group (10 s) was found in the 1 W group (4.28 ± 1.80 MPa), and the highest SBS mean value of 12.13 ± 3.30 MPa was found in the 2 W group.

As summarized in Table 5, statistically significant difference in SBS was found between the 1 W laser group and the 2 W laser group (*P* < 0.001), the 1 W laser group and 2.5 W laser group (*P* < 0.001), and the 1 W laser group and the acid-etched group (*P*=0.001). However, no significant difference in SBS was found between the other laser groups with one another and between the other laser groups with the acid-etched group.

### 3.2. ARI Values

The ARI scores are listed in (Table 6). The chi-squared test revealed that no statistically significant difference was found among the groups (*P*=0.059). Most of the samples had ARI type 2 or 3.

### 3.3. SEM

**Figure 5:**
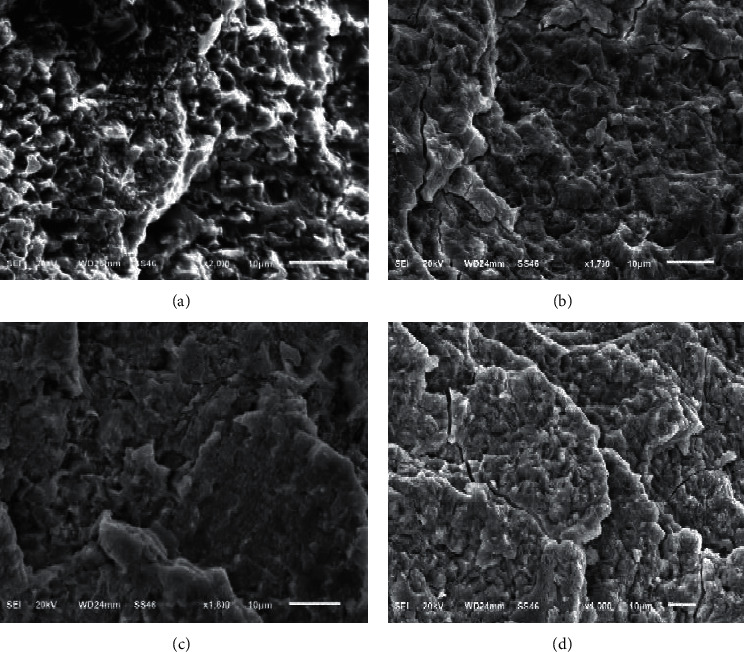
SEM images of laser-etched samples irradiated for 5 s at 1 W power (a), 1.5 W power (b), 2 W power (c), and 2.5 W power (d).

Figure 5 displays the SEM images of enamel surfaces exposed to different laser outputs (1, 1.5, 2, and 2.5 W) for 5 s. The effects of laser irradiation on group A at 1 W output and group B at 1.5 W output were insufficient to etch the enamel with a type III etching pattern, as described by Silverstone et al. [23]. However, irradiation of group C at 2 W output was sufficient to etch the enamel with an indiscriminate-type etching pattern. Irradiation of group D at 2.5 W output with a type III etching pattern was associated with some subsurface cracks.

**Figure 6:**
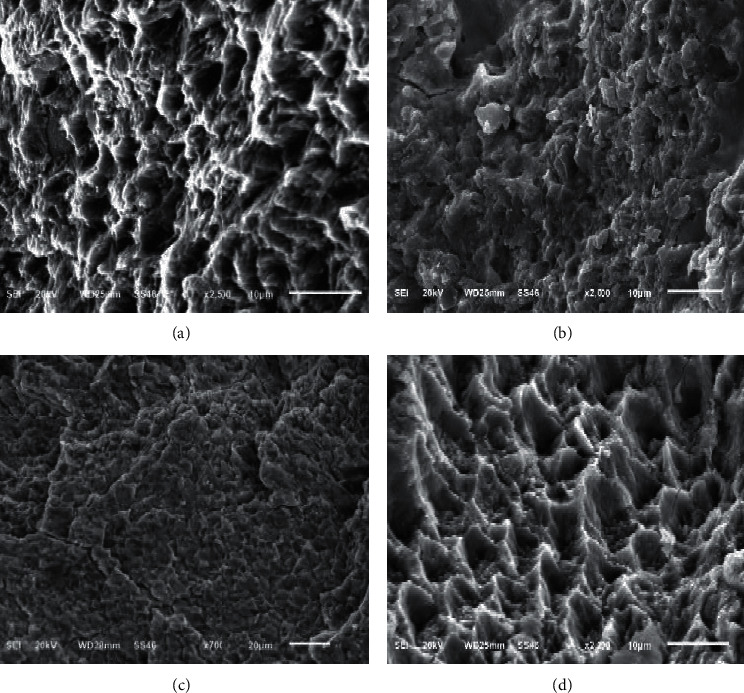
SEM images of laser-etched samples irradiated for 10 s at 1 W power (a), 1.5 W power (b), 2 W power (c), and 2.5 W power (d).

Figure 6 displays the SEM images of specimens irradiated with different laser outputs (1, 1.5, 2, and 2.5 W) for 10 s. In group A at 1 W output, it showed that the procedure was not enough to etch the enamel with dispersed honeycombs or craters. In group B at 1.5 W output, it observed that the procedure was enough to etch the enamel with a type III etching pattern; the same result was found in group C at 2 W and group D at 2.5 W with a type I etching pattern.

**Figure 7:**
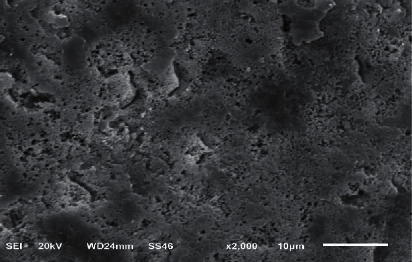
SEM image of the sample etched by phosphoric acid.

The enamel surface etched with phosphoric acid revealed a rough surface with numerous little grooves and pits, similar to a type III etching pattern (Figure 7).

## 4. Discussion

In the present study, the effects of Er, Cr: YSGG laser radiation for short periods or phosphoric acid etching on the bonded brackets' SBS, surface texture, and ARI scores were evaluated.

Maleic and polyacrylic acids have been used to avoid enamel loss due to demineralization by phosphoric acid (37%) during the bracket bonding process. However, the use of maleic and polyacrylic acids has resulted in a decrease in bond strength [26, 27]. Lasers, such as CO_2_, Nd: YAG, diode, and argon, induce caries resistance and are very essential in orthodontics, but the use of these lasers on dental hard tissues causes thermal damage, thereby making these lasers inappropriate for hard tissue treatments [28].

Recently, the Er, Cr: YSGG hydrokinetic laser that contains a cooling system (a mixture of air and water vapor) and does not involve vibration or heat has become one of the most widely used lasers for reducing enamel demineralization [29]. Moreover, the thermal ablation produced by Er, Cr: YSGG laser irradiation has been sufficient to alter the crystallinity and composition of enamel (e.g., the removal of carbonate, creation of new crystalline phases, and the increase in the hydroxyapatite minerals), and these changes may explain why laser-etched enamel is more resistant to the early development caries [30].

The appropriate time for acid etching ranged from 15 s to 60 s. Barkmeirer et al. [31] showed that the proper time for acid etching using phosphoric acid (37%) for good adhesion in orthodontic bonding was 15 s. In most of the previous studies, Basaran et al. [14] and Arora et al. [32] used the erbium lasers (Er,Cr:YSGG or Er,YAG) to etch the enamel surface with different laser outputs and constant time 15s, in accordance with the time required for etching enamel with phosphoric acid (37%).

To our knowledge, no previous studies investigated the SBS of bonded brackets using the Er, Cr: YSGG laser at different outputs and short periods of time 5 and 10 s. The result of the current study found that the SBS for the acid-etched group was 10.28 ± 5.12 MPa, which is adequate for proper adhesion strength. The null hypothesis can be rejected as the SBS in the laser irradiation 1 W group at 5 s and 10 s was 2.31.85 and 4.28 ± 1.80 MPa, respectively; which is clinically unacceptable and below the mean SBS of the acid-etched group. This finding is in agreement with the study by Berk et al. [13] and Usmez et al. [33], who compared the SBS of brackets using the Er, Cr: YSGG laser 1 W for 15 s and found that the SBS was significantly less than acid etching. However, our findings disagree with those of Basaran et al. [15], who evaluated the SBS after pretreatment by the Er, Cr: YSGG laser (operated at 1 W or 2 W for 15 seconds) and found that the SBS was comparable to that obtained with acid etching.

In addition, the SBS in the laser irradiation of 1.5 W groups at 5 s was 5.89 ± 1.84 MPa, which is lower than the SBS of the acid-etched group. The null hypothesis can be accepted as the SBS value at 1.5 W for 10 s was 8.98 ± 4.98 MPa, which was inferior to SBS achieved by the acid-etched group, but the difference was not statistically significant, and it is clinically acceptable and enough to etch the enamel, which is in agreement with a previous study of Basaran et al. [34] who found that the SBS at 1.5 W and 1.75 W at 15 s was adequate for bond strength.

In addition, the SBS values obtained with laser treatment at 2 and 2.5 W for 5 s were 10.648.70 and 9.45 3.23 MPa, respectively, which were clinically acceptable and similar to the mean SBS of acid-etched samples. Whereas the SBS value at 2 W for 10 s was 12.13 ± 3.30 MPa, which was the highest SBS obtained in this study and was greater than the SBS value of the acid-etched group; however, the difference was not statistically significant, followed by the SBS value obtained at 2.5 W for 10 s (11.65 ± 7.04 MPa), which was clinically acceptable and comparable with the mean SBS value obtained from acid-etched samples. This agrees with the study of Aroa et al. [32], who found SBS achieved by laser etching at 2 W and 2.5 W power for 15 s was comparable to phosphoric acid etching, but in a scanning electron microscope in 2.5 W, it was noticed that there is more destruction of the enamel.

The SEM analysis showed that the acid-etched sample had visible uniform and slight grooves. However, the laser-etched groups showed even surfaces when treated at low power output (1 W for 5 and 10 s); thus, these treatments were not suitable for etching the enamel with few microcracks. Irradiation at high power outputs (2 and 2.5 W) for 5 and 10 s allowed the etching of the enamel with uneven and heterogeneous surface and resulted in a preferable etching pattern.

Bond failure within the adhesive or at the bracket-adhesive contact was preferable to failure at the enamel-adhesive interface, which could result in enamel fracture and could risk crack reduction when debonding, according to the study of Bishara et al. [35]. In this study, the majority of the ARI scores were 2 or 3 in both acid and laser groups, indicating that the specimens had bond failure sites at the bracket-adhesive interface. However, more time was needed to remove the remaining adhesive [36], which can damage the enamel surface during the cleaning process [37].

In vitro studies have inherent limitations because they cannot entirely replicate clinical conditions such as intraoral contamination, moisture, temperature, and other variables such as mastication forces, trauma, and orthodontic mechanics, all of which have been found to affect bond strength [38]. Although this study was conducted on extracted human premolars teeth rather than bovine teeth, which have been shown to dissolve two to three times faster than human enamel [39], and the teeth were subjected to thermocycling to imitate the heat and humidity conditions of the oral cavity, it is nearly impossible to replicate all oral environment factors, which is a limitation of our study. Hence, it is recommended to conduct in in vivo studies to test the failure rate of orthodontic brackets bonded following Er. Cr: YSGG laser irradiation.

## 5. Conclusion

The laser irradiation at 2 and 2.5 W for 5 s was similar to that produced by acid etching, whereas the laser irradiation at 2 and 2.5 W at 10 s was higher compared with that obtained with acid etching and adequate to etch the enamel.

The use of Er, Cr: YSGG laser for short periods of time (5 and 10 s) is faster than acid etching and helps maintain the integrity of the teeth's surface.

## Figures and Tables

**Table 1:** Definition of laser groups.

Groups	Experimental procedure
Group 1 (*n* = 12)	Control group etched with 37% phosphoric acid
Group 2 (*n* = 12)	Enamel conditioned with the Er, Cr: YSGG laser at 1 W power
Group 3 (*n* = 12)	Enamel conditioned with the Er, Cr: YSGG laser at 1.5 W power
Group 4 (*n* = 12)	Enamel conditioned with the Er, Cr: YSGG laser at 2 W power
Group 5 (*n* = 12)	Enamel conditioned with the Er, Cr: YSGG laser at 2.5 W power

**Table 2:** Definition of laser groups regarding times.

	5 s (*n*)	10 s (*n*)	
Group 2	6	6	Enamel conditioned with the Er, Cr: YSGG laser at 1 W power
Group 3	6	6	Enamel conditioned with the Er, Cr: YSGG laser at 1.5 W power
Group 4	6	6	Enamel conditioned with the Er, Cr: YSGG laser at 2 W power
Group 5	6	6	Enamel conditioned with the Er, Cr: YSGG laser at 2.5 W power

**Table 3:** Descriptive statistics of different laser and acid etching groups (MPa).

Method	*N*	Mean	SD	Minimum	Maximum
1 W	12	3.30	1.69	1.25	7.38
1.5 W	12	7.44	3.93	4.16	18.20
2 W	12	11.39	6.32	3.77	26.66
2.5 W	12	10.55	5.35	4.21	23.52
Acid etching	12	10.28	5.12	3.29	20.99

**Table 4:** SBS (mean ± SD) of different laser etching methods according to time.

Method (W)	5 seconds	10 seconds
	Mean ± SD	Mean ± SD
1	2.31 ± 0.85	4.28 ± 1.80
1.5	5.89 ± 1.84	8.98 ± 4.98
2	10.64 ± 8.70	12.13 ± 3.30
2.5	9.45 ± 3.23	11.65 ± 7.04

**Table 5:** Pairwise comparisons between the different etching methods.

Method	1 W	1.5 W	2 W	2.5 W	Acid etching
1 W	—	*P*=0.090	*P* < 0.001	*P* < 0.001	*P*=0.001
1.5 W	—	—	*P*=1.000	*P*=1.000	*P*=1.000
2 W	—	—	—	*P*=1.000	*P*=1.000
2.5 W	—	—	—	—	*P*=1.000
Acid etching	—	—	—	—	—

*P* ≤ 0.05 significant.

**Table 6:** Association between ARI scores and the different etching groups.

Method	ARI				*χ*2	*P*
	0	1	2	3		
1 W	0 (0.0)	1 (8.3)	6 (50.0)	5 (41.7)	20.44	0.059
1.5 W	0 (0.0)	3 (25.0)	3 (25.0)	6 (50.0)		
2 W	0 (0.0)	5 (41.7)	5 (41.7)	2 (16.7)		
2.5 W	0 (0.0)	1 (8.3)	7 (58.3)	4 (33.3)		
Acid etching	2 (16.7)	0 (0.0)	4 (33.3)	6 (50.0)		
Total	2 (3.3)	10 (16.7)	25 (41.7)	23 (38.3)		

*P* ≤ 0.05 significant.

## Data Availability

The data supporting the findings of the study are available from the corresponding author upon responsible requests.
